# Service provider perspectives on implementing the ‘F-words for child development’: an innovative approach to children’s health and developmental services

**DOI:** 10.3389/fpubh.2026.1788060

**Published:** 2026-09-18

**Authors:** Isha Mehreen, Vanessa Tomas, Evelyn Bursey, Lucy Charles, Andrea Cross, Arul Hamill, Elizabeth Hammond, Kim Hesketh, Julia Hill, Brigid Hitchcock, Margaret Jones, Genevieve McMurdie, Peter Rosenbaum, Rachel Teplicky, Margaret van Beers, Kristy Wittmeier

**Affiliations:** 1 CanChild Centre for Childhood-onset Disability Research, McMaster University, Hamilton, ON, Canada; 2 Department of Pediatrics, McMaster University, Hamilton, ON, Canada; 3 Community Living North Halton, Milton, ON, Canada; 4 Paediatric Occupational Therapy and Physiotherapy, Auckland, New Zealand; 5 Rehabilitation Centre for Children, Winnipeg, MB, Canada; 6 Department of Physical Therapy, University of Manitoba, Winnipeg, MB, Canada; 7 Children’s Treatment Network, Richmond Hill, ON, Canada; 8 Department of Physiotherapy, Auckland University of Technology, Auckland, New Zealand; 9 Active Living and Rehabilitation, Aotearoa, New Zealand; 10 Department of Occupational Science and Therapy, Auckland University of Technology, Auckland, New Zealand; 11 Children’s Developmental Services Halton Region, Oakville, ON, Canada; 12 Department of Pediatrics and Child Health, University of Manitoba, Winnipeg, MB, Canada

**Keywords:** childhood-onset disability, F-words for child development, ICF, implementation, service provider experiences

## Abstract

**Background:**

The ‘F-words for Child Development’—Functioning, Family, Fun, Friends, Fitness and Future—provide a framework that brings to life key World Health Organization ideas about child health and development in an innovative and family-friendly way. Given international uptake of the F-words, understanding service providers’ (SPs’) experiences and perceived impact of F-words on pediatric disability and developmental services is important.

**Objectives:**

This study explored SPs’ (i) knowledge of the F-words and confidence in using the framework; (ii) perceptions and experiences using F-words; and (iii) barriers/facilitators to implementing the F-words.

**Methods:**

This descriptive study was part of a larger project implementing the F-words across five sites in Canada and New Zealand from 2021 to 2024. SPs who provide services for children and youth with disabilities or developmental concerns (e.g., neurodevelopmental disabilities) were surveyed and interviewed after F-words implementation to explore their experiences implementing the F-words. Quantitative data were analyzed using descriptive statistics, and qualitative data were analyzed via team-based conventional content analysis.

**Results:**

Fifty-seven SPs completed the survey and 10 participated in interviews. SPs rated F-words familiarity (X̄ = 3.41/4) and knowledge (X̄ = 3.05/4) high, with moderate confidence using the F-words (X̄ = 2.93/4). Content analysis revealed that SPs perceived the F-words to enhance their service-delivery experiences, indicating that they provide a structured yet flexible approach to deliver strengths-based, holistic, family-centered care, while fostering relationship-building and empowerment with/among families. Examples of implementation facilitators included organizational buy-in, access to F-words resources/tools, and F-words alignment with service-delivery models. Reported implementation barriers included time constraints, the need for ongoing F-words training, and uncertainties regarding the cultural/social safety and responsiveness of the F-words for families.

**Conclusion:**

Within this study, SPs had positive perceptions and experiences using the F-words to promote strengths-based, family-centered care for children and youth with disabilities or developmental concerns and their families. Future efforts should address implementation challenges, leverage facilitators, and continue to explore F-words implementation experiences across diverse settings.

## 1 Introduction

Neurodevelopmental disabilities (NDDs) affect up to 17% of children in North America (1) and have lifelong implications for a child’s functioning, participation, and family well-being (2, 3). Children and adolescents with NDDs such as autism, cerebral palsy, intellectual disabilities, and learning disabilities and their families frequently navigate health, education and social systems that are fragmented, deficit-focused and often not aligned with their realities and lived experiences (4–7). *Traditional* models of care that support these children and their families have historically emphasized impairments and limitations, which can marginalize children and caregivers, reinforce disempowerment, and contribute to unmet needs in service delivery (5, 8, 9).

In response to these challenges, a significant transformation has occurred in child developmental services over the past few decades, shifting beyond a medical model toward a broader, more holistic, biopsychosocial approach (10). The medical model primarily focuses on diagnosing and remediating impairments, often overlooking broader contextual factors such as environmental, personal, and social influences (11). In contrast, the biopsychosocial model integrates biological, psychological and social dimensions of health, recognizing the complex interplay among a child’s health condition and personal factors, and emphasizing daily functioning rather than solely the presence of a health condition (12, 13).

This paradigm shift is reflected in the World Health Organization’s International Classification of Functioning, Disability and Health (ICF), which provides a framework for health and is key to informing modern, pediatric care (14). The ICF promotes a holistic perspective, extending beyond medical diagnoses to encompass functioning, participation, and the influence of environmental and personal factors. It functions as a comprehensive framework for understanding and documenting functioning and disability, classifying health-related domains in terms of *body functions and structures*, *activities* and *participation*, and *contextual factors*. The ICF has been increasingly adopted in research and practice to guide assessment, intervention and service delivery, fostering a shared language across health, education and social services (15).

The ‘F-words for Child Development’, developed by Rosenbaum and Gorter, operationalize the ICF into six accessible ‘F-words’: Functioning, Family, Fitness, Fun, Friends and Future (9). These F-words encourage a holistic, strengths-based, function-focused, family-centered and lifespan approach to health and child development (16, 17). Rather than focusing on limitations, the F-words framework and its associated tools invite service providers (SPs) and families to explore collaboratively the child’s strengths and what matters most to the child and family, fostering service-delivery that’s reflective of their priorities and context (16, 17). This approach challenges biomedically-centered and deficit-based methods—still prevalent in healthcare systems, particularly in rehabilitation and disability services—by leveraging strengths, promoting a person’s functioning, and considering the broader context of their life (16, 17). In doing so, the F-words translate the ICF’s concepts into everyday language and practice. Since 2012, the F-words have gained international recognition as an innovative and impactful approach guiding clinical practice, research and policy (18). Their global uptake is evident through more than 35 language translations of the original F-words concepts (e.g., Spanish, Urdu, Arabic, Vietnamese) (19); over 100,000 unique international visitors to the F-words Knowledge Hub (20);^1^ and >3,600 graduates of the F-words Online Training Program (21). International impact is evident through research and implementation activities across Canada, New Zealand, Iran, Brazil, and Australia (22–26). Studies have indicated that the F-words promote a strengths-based approach, empower families, encourage a holistic and balanced clinical assessment, and support meaningful goal setting (18, 22, 24, 27).

Despite the global interest in the F-words, the integration of the F-words into health service delivery is an evolving process. Thus, understanding how the F-words are being implemented, adapted and experienced by interest holders and users such as SPs is essential to realizing their full potential in improving outcomes for children and adolescents with NDDs and their families. To date, studies have explored how the F-words are used by SPs, such as in rehabilitation (18, 28), oncology (29), and neonatology (30) contexts. There has yet to be an in-depth exploration of F-words implementation at an organizational level (e.g., the barriers, facilitators, implementation strategies used) and the experiences of SPs participating in implementation. Additionally, understanding the impact of the F-words on SP practice and relationships with families is lacking in the literature. This manuscript reports research that was embedded within a larger F-words implementation study that aims to address these gaps.

### 1.1 Objectives

This descriptive study had the following two research objectives:

1. Understand SPs’ knowledge, attitudes toward, and use of the F-words and assess their perceived impact on service delivery and family-centered care.
2. Explore SPs’ perspectives on the influence of contextual factors (e.g., barriers and facilitators) on F-words implementation.

## 2 Methods

### 2.1 Project context

This study was part of a larger project that explored and evaluated the implementation of the F-words across five developmental services organizations from 2021–2024: three in Ontario, Canada, one in Manitoba, Canada, and one in Auckland, New Zealand (Aotearoa). The larger project was led by CanChild’s F-words Team (researchers, families, clinicians), clinical and leadership partners from the five organizational sites, and clinician researchers from the Auckland University of Technology. Following a convergent parallel mixed methods, hybrid type III effectiveness-implementation, pre-post pilot design (31), the purpose of that larger project was to explore: i) how the F-words are implemented at an organizational level, the factors that influence implementation, and the acceptability, feasibility, and appropriateness of F-words implementation; and ii) the impact of F-words implementation on, and experiences of, SPs and families. Over approximately 2 years (varied across sites), each organization rolled out an F-words implementation plan. This paper reports on SPs’ experiences using, and impacts of, F-words (research objective #2). A separate manuscript on the evaluation of F-words implementation is under review,^2^ and the manuscript on families’ experiences is reported elsewhere (32). There is no data overlap between these three manuscripts.

### 2.2 Participants and data collection

The five participating organizations provide a range of services for children and youth with disabilities and developmental concerns (e.g., NDDs), and their families; examples include assistive technology, occupational therapy, physiotherapy, speech and language services, coordinated service planning, and inclusion services. Any SP (e.g., occupational therapist, physiotherapist, resource consultant, service navigator) employed at the organization during the time of the study could participate. Recruitment strategies included emailing SPs with an email script, distributing the study flyer via email, sharing study information during staff meetings and presentations, and posting study information on social media platforms. Ethics approval was obtained from institutions in Ontario and Manitoba, Canada (2021), and New Zealand (2022).

Data collection comprised surveys and online interviews. Survey questions included open-ended questions on F-words implementation barriers and facilitators and overall perceptions of the F-words; and self-developed 4-point Likert-scaled questions about perceived knowledge of F-words, familiarity, attitudes toward using the F-words, and confidence using the F-words (e.g., not at all confident, somewhat confident, confident, very confident). Likert-scaled questions were developed with researcher, clinician, and family input among the project team. We also asked whether participants have used the F-words (options: yes, no, no answer). Interview questions asked about how SPs use the F-words, barriers to and facilitators of their use, and their perceptions on their impact (Supplementary file 1). The surveys were completed, and interviews were conducted through McMaster University’s secure REDCap and Zoom platforms, respectively. Informed consent was obtained through REDCap separately for the surveys and interviews. Timing for interviews and surveys was dependent on organizations’ implementation plans, and when they entered the ‘post-implementation’ phase of the study. Surveys were completed between late 2022 and early 2024. Interviews were conducted by the first author, second author, and a research assistant in 2023 for the Canadian organizations, and early 2024 for the New Zealand organization. The second author has experience with the F-words, qualitative research, and implementation science, and provided support and guidance to the first author and research assistant when they conducted interviews. Interviews were recorded via Zoom, transcribed through Zoom’s auto-transcription feature, and reviewed, cleaned, and de-identified by the first author.

### 2.3 Data analysis

For quantitative survey data, we conducted a descriptive statistical analysis using Microsoft Excel. For qualitative data, Hsieh and Shannon’s approach to conventional content analysis was used (33). Rather than using existing theory (or predefined assumptions about F-words) to develop codes, we derived codes directly from the interview and open-ended survey data and then constructed descriptive categories. However, we acknowledge our team’s pre-conceptions regarding benefits of F-words (e.g., strengths-based, family-centered), due to our experiences with F-words and anecdotal evidence. We explicitly identified and documented these assumptions and remained open to perspectives that might challenge or extend our existing understanding. During analysis, no prior empirical evidence existed to inform our expectations regarding barriers and facilitators to implementing F-words in these contexts.

To ensure consistency and shared understanding, the first and second authors collaboratively analyzed three interview transcripts to establish the initial coding framework and operational definitions. The first author then led the coding of the remaining transcripts, meeting with the second author to discuss questions, refine the coding framework, and resolve discrepancies. The second author reviewed all coded transcripts and provided feedback. For survey data, the first author applied the same coding framework with feedback from the second author. The list of codes, along with their definitions and illustrative quotes, were circulated to the research team for feedback and discussed during a meeting. The first author then organized the codes into categories and sub-categories, incorporating feedback from the larger team. The finalized categories were shared with the research team for additional feedback and validation of interpretation. Please note that we did not conduct additional analyses to compare quantitative and qualitative data directly, as data were used to provide a descriptive overview. Our team reflects on the quantitative and qualitative data in the discussion of this manuscript.

Recognizing that some members of the research team are connected to F-words (e.g., their development, support implementation), to mitigate confirmation bias, receiving feedback on codes, having group discussions, and engaging in ongoing reflexivity were imperative. The first and second author kept an audit trail of decision points and questions/reflections throughout the process and actively engaged in reflexive discussion to explore how experiences and expectations (e.g., F-words as strengths-based) may influence coding and interpretation. Regular discussions among our team challenged individual interpretations to consider multiple perspectives, ensuring interpretations were grounded in participants’ experiences.

## 3 Results

Fifty-seven SPs completed the post-study survey, and there were no missing data for the non-demographic items reported in this manuscript. Ten SPs also participated in an online interview. See Table 1 for participant demographics. SPs identified working in a variety of programs/services, such as coordinated service planning, inclusion and coaching services, feeding clinics, school-based rehabilitation services, early intervention services, diagnostic autism clinical services, and fetal alcohol syndrome disorder programs. Overall, 51 (89%) SPs reported having used the F-words in their programs/services.

**Table 1:** SP participant demographics.

Demographic category	N (%)
Gender	
Woman	55 (96%)
Man	1 (2%)
No response	1 (2%)
Age	
26–30 years	4 (7%)
30–35 years	9 (16%)
35–40 years	7 (12%)
40–45 years	12 (21%)
45–50 years	9 (16%)
>50 years	14 (25%)
No response	2 (4%)
Profession	
Occupational Therapist	12 (21%)
Consultant Roles (i.e., resource consultant, developmental service consultant)	11 (19%)
Physiotherapist	9 (16%)
Speech Language Pathologist	8 (14%)
Coordinator	4 (7%)
Service Navigator	4 (7%)
Other (e.g., social worker, dietitian)	6 (11%)
No response	3 (5%)
Time in field	
< 5 years	7 (12%)
5–10 years	13 (23%)
10–20 years	14 (25%)
20–30 years	14 (25%)
30 + years	8 (14%)
No response	1 (2%)
Organization location	
Ontario, Canada	34 (60%)
Manitoba, Canada	17 (30%)
Auckland, New Zealand (Aotearoa)	6 (11%)

The 10 SPs who participated in the post-study interview were from Ontario (*n* = 4) and Manitoba (*n* = 3), Canada, and Auckland, New Zealand (*n* = 3). Professions included physiotherapists (*n* = 4), resource consultants (*n* = 3), occupational therapists (*n* = 2), and one speech-language pathologist (*n* = 1). Interview duration ranged from 26 to 46 min.

Table 2 displays the mean scores (standard deviations) of SPs’ rated familiarity with the F-words and knowledge, confidence, and thoughts on using the F-words. Overall, most SPs rated F-words familiarity high [‘very familiar’ (54%) or ‘familiar’ (35%)]. Additionally, 37% of participants ‘extremely liked’ and 51% ‘liked’ the F-words. For knowledge, 50% of SPs selected being ‘knowledgeable’ about the F-words, while 29% and 20% selected being ‘very knowledgeable’ and ‘somewhat knowledgeable’, respectively. For confidence, 46% felt ‘confident’ in their ability to use the F-words, while 26% felt ‘very confident’ and 23% felt ‘somewhat confident.’

**Table 2:** SP perceptions of using the F-words (*n* = 57).

Statement with scales	4-point Mean ± SD
1. How FAMILIAR are you with the F-words? (1 = not at all familiar, 2 = somewhat familiar, 3 = familiar, 4 = very familiar)	3.41 ± 0.73
2. What is your level of KNOWLEDGE of the F-words? (1 = not at all knowledgeable, 2 = somewhat knowledgeable, 3 = knowledgeable, 4 = very knowledgeable)	3.05 ± 0.75
3. How CONFIDENT are you in your ability to USE the F-words? (1 = not at all confident, 2 = somewhat confident, 3 = confident, 4 = very confident)	2. 93 ± 0.84
4. What are your THOUGHTS on the F-words? (1 = dislike the F-words, 2 = no opinion either way, 3 = like the F-words, 4 = extremely like the F-words)	3.21 ± 0.75

For qualitative data, six categories were developed. The first four categories address F-words implementation barriers, facilitators, and F-words use, and categories five and six offer insights into SPs’ experiences and perceptions of impact.

1. Use and Uptake of the F-words
2. “It’s still quite challenging”
3. Culture- and Context-Specific Challenges
4. Implementation Opportunities
5. Structured Yet Adaptable Framework
6. The F-words may Enhance Service Provider Experiences

### 3.1 Category 1: use and uptake of the F-words

The F-words have been applied in different ways. This includes intake processes, information gathering, goal setting, transition planning (e.g., transition into school, out of school), embedding F-words into documentation/reports, and F-words educational materials (e.g., newsletters, introductory sheets for families, staff profiles displayed in clinics). The three most frequently identified examples are detailed below.

Numerous SPs shared that they use the F-words to set goals collaboratively with children and families. The F-words can support families to identify and prioritize meaningful goals. One SP from Ontario (interview) described how they supported a family in developing and prioritizing goals using the F-words:

*There was a bubble chart with the [F-words] and the goals, so I shared that with this family, and I asked her, ‘I know there are so many goals that you want to work on, but obviously we cannot work on 10 goals at the same time, so we want to pick two’. So, I sent her that document, [and asked] choose your two priority areas that you want to work on and then have one goal for each priority area’.*

Several SPs described embedding the F-words into their documentation and reports. One SP from New Zealand (interview) shared how the F-words and the F-words Life Wheel, an F-words coaching tool created in New Zealand (34), provide a structured way to document a child’s progress:

*Our report follows the structure of F-words and we have got some goals there. We might do an intervention of six sessions, and then we review, after a period of consolidation, maybe three months later. Then I pull up the reports, and I go through the report with the family in terms of this is where your child was at. can you tell me what it looks like now?*

The F-words were also used to support and facilitate transition periods:

*I also use it in transitions, whether that child’s transitioning to school, or transitioning into a daycare or transitioning to a new place. It was really helpful for a child that was homeschooled up until the age of 7, and that was a child with Down syndrome, and we did an F-words [profile] for that initial Individualized Education Plan planning meeting when she was transitioning from being homeschooled to then going to a public school.* (Manitoba, interview).

### 3.2 Category 2: “It’s still quite challenging”

Despite innovative and diverse applications of the F-words, SPs still encounter challenges in conceptualizing and using the F-words. This category comprises two subcategories: (i) *conceptual challenges with specific domains* and (ii) *challenges in operationalization*.

#### 3.2.1 Conceptual challenges with specific domains

Some SPs found it difficult to engage families in discussions about ‘Future’, particularly when families struggled to envision a positive outlook for their child. This challenge was emphasized by SPs when working with families who identify limited perceived progress. As one SP explained:

*But it’s just that ‘future’ can really confuse some people when they do not see a positive future for their child just yet. All they see are those drawbacks, and we are hitting a brick wall kind of thing, where they are not improving with their skills, so it’s hard to see the future.* (Ontario, interview).

Similarly, SPs encountered challenges with ‘Functioning’, which was perceived as broad and difficult to operationalize. This sometimes led to a tendency to focus on deficits, making it difficult for SPs to maintain a strengths-based focus. SPs shared that they had to focus intentionally on how the child can use their strengths to participate in everyday activities.

*For the function section, I need to constantly remind myself that it is not meant to be about what can you do and what cannot you do (*i.e.*, as a way of identifying needs for treatment) but rather how the client uses their strengths to participate in functional tasks.* (Ontario, survey).

#### 3.2.2 Challenges in operationalization

A few SPs described challenges in using the F-words due to factors such as limited time, documentation requirements, competing priorities, and a lack of standardization in F-words use across programs and services.

For example, some SPs faced barriers to using the F-words in the context of current, deficit-based service systems and what families are accustomed to (i.e., medical model, therapist-driven). SPs also discussed difficulties aligning the F-words with institutional documentation practices and professional requirements. There was a noted inconsistency of how F-words are used across programs and partner organizations/agencies. One SP (Ontario, interview) shared:

*Some of the challenges with the F words are for those families who have a huge clinical team [.] They’re going to different agencies and organizations to support a lot of those developmental pieces. Their documentation is still very clinical, like the things that they are working on, and then, we are taking such a different approach.*

SPs also shared that some of their colleagues are unsure whether the F-words adequately capture essential information needed for their practice. One SP (Manitoba, interview) shared feedback they received from colleagues:

*We have a guiding question sheet that we want people to fill out that serves as their initial paperwork when we see them, instead of charting. And some of our clinicians were very like ‘well, I’m not going to be able to capture what I really want to capture’. We always say this is just a guiding framework - you can write anything down you want. [.] We always say that these conversations can happen while you are working with the child.*

### 3.3 Category 3: cultural and context-specific considerations

SPs identified considerations and challenges when using the F-words with families who are experiencing critical and/or crisis situations. As one SP (New Zealand, interview) explained:

*More complex scenarios of families with maybe limited capacity in terms of if they are in crisis, I think can be quite difficult. Families where they have children who are nonverbal, any kid there has got really complex behaviors is quite tricky, because things naturally always gravitate to the negatives.*

Other SPs highlighted challenges when working with underserved and equity-deserving communities. Some SPs shared examples of families with limited English proficiency or unstable living conditions who may struggle to engage meaningfully with the framework:

*A lot of times we work with newcomers, and we also have a very marginalized population in our downtown area where the social situations are just. they are devastating. So, trying to put the F-words into something where we are not even quite sure [about family safety/security], I think that sometimes it is when we hear about some of those challenging conversations that it comes to that.* (Manitoba, interview).

Some SPs discussed limitations of the F-words specific to cultural considerations, noting that important constructs may not be captured, such as spirituality. A few SPs raised concerns about the connotation of the term “F-words” and its potential to be misunderstood or perceived as culturally inappropriate. To address this, some opted for alternative phrasing, such as “Our Favorite Words,” to make the framework more accessible across diverse populations.

Moreover, in contexts where services are privatized, SPs noted that families’ perceptions of value for money could affect their willingness to engage with the F-words. In New Zealand, for example, one SP emphasized the influence of privatization:

*The goal setting varies with each family and their acceptance of a session around goal setting. [.] Sometimes I change my way of implementing it, if I suspect that the family is going to walk out of there and think* ‘well, that wasn’t value for money because I didn’t do Physio.’ *So, there is still that mindset that we have got to work around with families.* (NZ, interview).

### 3.4 Category 4: implementation opportunities

Two subcategories comprise this category: (i) *supports and facilitators* and (ii) *barriers and challenges*. SPs shared insights on facilitators and barriers to implementation of the F-words in their practice.

#### 3.4.1 Supports and facilitators

Several SPs highlighted supports that facilitated F-words implementation at the organizational level. These included alignment with existing service delivery models, organizational buy-in, and access to F-words resources and training.

SPs noted that the F-words complemented existing approaches to service delivery (e.g., family-centered service, solution-focused coaching), making it easier to integrate into existing workflows. Organizational support was another facilitator. Many SPs identified champions within their organizations, such as managers and colleagues, who played a key role in driving implementation. SPs also emphasized that leadership support helped create a foundation for change, facilitated collaboration between teams and agencies, and enabled necessary adjustments to documentation.

*Support at a program level is really helpful in allowing you to implement things at an individual level. [.] none of the documentation changes that we have made, or that we are hoping to make, would have happened without support at the management level.* (Manitoba, interview).

Access to F-words resources and training was also noted as essential. Many SPs referenced the CanChild website, the original F-words paper, and F-words videos as valuable tools. Additionally, some organizations developed internal resources, such as workshops and trainings. One SP shared how an expanded list of F-words prompts/guiding questions developed by their organization was beneficial in guiding discussions with families:

*I did get [a] more expansive list of questions. I use that to help me, because if I ask about function, sometimes the parents do not know how to answer without the guidance of sub-questions. It’s nice to have a more in-depth guideline.* (Ontario, interview).

#### 3.4.2 Barriers and challenges

SPs also identified barriers to implementation at individual and organizational levels. Time was a major challenge; with many providers indicating they struggled to incorporate the F-words into their appointments. One SP noted:

*I do not always have the time or foresight to have my clients fill out the F-Words worksheet. I am often trying to make my appointments as efficient as possible, because they can often be long and tedious. (Manitoba, survey).*

Some SPs also encountered resistance from colleagues who preferred ‘traditional’ assessment methods: S*ome colleagues do not want to use it and still prefer the ‘old-school’ method of having families list all their concerns in a rigid intake form.* (Manitoba, interview).

One SP emphasized the need for continuous training, noting that periodic refreshers could help maintain engagement and deepen understanding: *Training every five years or so is not really enough to build skills in understanding/using this tool effectively.* (Ontario, survey).

### 3.5 Category 5: structured yet adaptable framework

SPs valued the F-words for providing a structured yet flexible approach to working with families. Several SPs appreciated how the F-words provide structure to guide their discussions with families, while also allowing for customization based on each family’s needs. SPs found that this structure enhanced accessibility by fostering a shared language between providers and families.

One SP from Manitoba (survey) expressed that the F-words made it easier to apply complex frameworks, like the ICF, in real-world settings, providing an important structure/framing:

*I like that the F-words provide a family friendly model for utilizing the ICF. In my Master’s program, the ICF was utilized as a key assessment tool for supporting clients and helping to determine goals. But I always found it hard to apply in real world situations with clients and families. The F-words provide a great template for working with families.*

In addition to offering structure, SPs emphasized the framework’s adaptability. Many found that its flexibility allowed them to integrate it into their diverse practices. One SP from Manitoba (interview) reflected on this, noting that while some colleagues viewed the framework as limiting, those who embraced its flexibility found it enhanced their work: *I just hope that people can see how it can be a flexible tool, and you can kind of make it what you need, and it’s not a super rigid. [.] We do not always fill out every section as full as the other section.*

### 3.6 Category 6: the F-words may enhance service provider experiences and practices

SPs shared how the F-words enhanced their approaches to service delivery, and the positive impact this has had. This category includes three sub-categories: (i) *The F-words facilitate authentic relationship-building with families*; (ii) *leverage family/child strengths and are holistic*; and (iii) *family-centered and empower families.*

#### 3.6.1 The F-words facilitate authentic relationship-building with families

SPs reported that the F-words helped them connect with the children and families on a more personal level. Participants noted that the F-words foster honest dialog, support communication on equal footing, and create a welcoming atmosphere, ultimately strengthening relationships with families: *I feel [F-words] have helped our services to have that authentic relationship with the parent, where the parent is open to us from the very first time and very honest* (Ontario, interview).

In establishing rapport, SPs found that families were more likely to trust them, leading to greater engagement with services and enhanced child/family relationships. One SP also emphasized how stronger connections improve overall outcomes: *If I connect with a family, I’m more likely to get feedback and parents listening to the things I’m suggesting and putting them through* (NZ, interview).

#### 3.6.2 Leverage family/child strengths and holistic approach

SPs highlighted that the F-words can facilitate a shift toward strengths-based and holistic services. Many SPs shared that this shift allowed them to support the child and family in achieving meaningful goals by focusing on individual abilities, aspirations, and well-being rather than limitations.

Another SP (NZ, interview) reflected on how adopting the F-words changed the way they engage with the child and family:

*I probably used to jump straight into the problem like, “What can I help you with today?” There’s so much more. There’s so many layers to it than just coming to me with not being able to jump or not being able to ride a bike.* [F-words approach] *given me a more holistic view of the child. Sometimes more often, it’s not always necessarily linked to why they have come to see me. We kind of use the F-words to unpack the different areas of the child’s day and life.*

#### 3.6.3 F-words are family-centered and empower families

SPs emphasized that the F-words reinforced and enabled them to take a family-centered approach to services. This shifts the focus from clinician-driven goals to goals that are meaningful for the child and family. Many SPs described how the F-words may empower families to take ownership of their care, define their priorities, and build their own capacity to drive decision-making.

Some SPs shared how the F-words facilitate rich discussions with families about their goals, allowing them to explore tailored approaches and solutions. One SP from Manitoba (survey) shared how using the F-words led to tailored strategies that aligned with what was most important to the family:

*I love that it reminds me to talk about what is important to the family. It leads to really rich discussions about the child and their goals. In some cases, it has led us to developing new and customized equipment solutions around what we learn about the family. For example, a family is not necessarily going to ask if we can make them a specialized switch adapted fishing pole, but when we find out that fishing is the most important activity to the family and that their child is not able to participate fully, we are able to offer it.*

## 4 Discussion

This study provides insights into SPs’ experiences implementing the ‘F-words for Child Development’, an innovative approach designed to operationalize the ICF framework. Familiarity of F-words was high and SPs shared positive experiences with F-words (e.g., F-words promote relationship-building, strengths-based services, family empowerment, flexibility of service planning); however, SPs equally highlighted important barriers and challenges (e.g., time constraints, differing mental models of disability services, conceptual challenges, cultural/social considerations). Learnings offer insights into how SPs may use the F-words in their practice, potential benefits, and challenges that warrant future exploration.

SPs viewed the F-words as a catalyst for a strengths-based, holistic, and truly family-centered approach. Participants felt that the F-words can empower families to define priorities, take ownership of care, and pursue meaningful goals by focusing on abilities and aspirations rather than limitations. This viewpoint has similarly been expressed by SPs in Australia (22) and Ireland (35), and families using the F-words in Canada (32), New Zealand (24) and Iran (25). Positive perceptions identified qualitatively converge with quantitative findings; specifically, participants indicating that they like or extremely like the F-words. Notably, unique to this study, SPs described their perceived stronger relationships with families when using the F-words. This is an important finding, suggesting that the F-words may serve as a communication and relational tool in clinical practice by building trust, fostering rapport, and supporting meaningful discussions.

Multiple enablers were identified that may support and expand use of the F-words, alongside challenges. The adaptability of the F-words is a particularly important finding. This can encourage organizations to tailor the F-words to their service models, approaches, and workplace processes, and SPs to adapt the F-words to meet families where they are. This flexibility may enable F-words to be used in diverse settings (36, 37), promote their future sustainability (38), and encourage their equitable use via intentional tailoring (39). It is also imperative to consider and address F-words implementation barriers. Reported challenges (e.g., time constraints, service restrictions, training needs) have recently been reported by SPs in Ireland, with a particular emphasis on resource and time constraints (35). These barriers also shed light on existing rehabilitation service system issues, such as over-worked clinicians and rigidity of service models (and accompanying documents and processes) that are not sensitive or responsive to emerging and unique family needs (40). Further, training for rehabilitation SPs may need to be better aligned with the philosophies underpinning the ICF and F-words. While examples are emerging of ICF in curricula (41–43), there is a need for more intentional, collaborative, tailored integration of ICF in higher education and with rehabilitation centers (44). To integrate ICF and F-words fully into service systems, we need to advocate for policy- and system-level changes. This is emerging in Ontario, Canada, evident through the Ontario Ministry of Children, Community, and Social Services embedding F-words into Preschool Speech and Language and Children’s Rehabilitation Service Guidelines (45). CanChild also offers a free Online F-words Training Program^3^ and a suite of paid training options^4^ that can be leveraged (21). Overall, these factors influencing implementation can be interpreted in relation to key theoretical constructs from the field of implementation science (e.g., organizational and systems-level contexts, adaptability, capability, and implementation processes), allowing for a cohesive explanation of how findings align with implementation science knowledge (i.e., Capability, Opportunity, Motivation - Behavior; Consolidated Framework for Implementation Research) (46).

A novel insight from this study was participants’ reflections on how cultural and social factors may influence use and reception of F-words. Some SPs noted that the F-words may not always resonate with families, particularly those facing complex diagnoses or crisis situations, from equity-deserving communities, and whose worldviews differ from Western perspectives. Importantly, these findings reflect SPs’ perceptions rather than direct evidence of limitations of the F-words. For example, the F-words are being used globally with culturally diverse communities [e.g., Brazil, Iran, Israel; (25, 27, 47)] and in historically under-resourced countries (23). Concerns expressed by SPs may reflect uncertainty regarding how to adapt and apply the F-words in diverse contexts, highlighting potential needs for resources, guidance, adaptations, and opportunities for critical reflection. Similar challenges were highlighted among SPs in Ireland who expressed difficulties navigating conservations about Future and Family with diverse families (35). CanChild’s F-words Team and international collaborators are pursuing work to improve cultural and social responsive applications of F-words. Work in New Zealand has suggested that more explicit consideration of spirituality and wellbeing may strengthen the relevance of F-words for some communities (24). CanChild’s F-words Team is also developing tools and resources to enable and illustrate responsive, flexible, and sensitive F-words use in diverse scenarios (e.g., ‘What If’ Tool).^5^

Interestingly, while participants rated F-words familiarity highly and positively perceived the F-words, they reported moderate levels of confidence using the F-words (i.e., only 26% selected ‘very confident’). This may indicate an ongoing challenge with integrating the F-words into their practice. Qualitative data helped explain this discrepancy, as participants described understanding and valuing F-words but facing challenges in applying them. This was due to uncertainties in how to use F-words in complex situations and resource-constrained contexts. This finding is consistent with implementation science theories, such as the Diffusion of Innovation Theory, which posits that awareness and positive attitudes toward an innovation often precede the development of confidence and habitual integration into practice (48, 49). Further, the Capability, Opportunity, Motivation – Behavior Theory (2.0) indicates that while someone may have the capability (e.g., familiarity and knowledge), without the motivation, which includes one’s degree of confidence, and opportunity (e.g., social supports, resources, time), the innovation may not be implemented or may be implemented inconsistently (50). Some SPs may be at earlier stages of adoption, demonstrating conceptual endorsement and persuasion, but require additional resources, opportunities, and support to move into consistent implementation.

### 4.1 Strengths and limitations

First, this study was conducted collaboratively with organizational leaders and clinicians, increasing the likelihood that analyses were grounded in practical realities and informed by diverse system-level perspectives. The inclusion of SP study participants from a wide range of professional roles, across five organizations, and two countries, enhanced the richness of the data, allowing the findings to reflect varied experiences in the NDD field. Finally, the study employed both quantitative and qualitative approaches, enabling a broader understanding of the factors influencing F-words implementation.

Study limitations should also be acknowledged. Since all SPs across organizations were invited to take part, some respondents may have been working in programs not directly involved in implementing the F-words as part of the larger implementation study. As a result, they may have been less familiar with existing activities and initiatives related to the F-words. Participation may also have been influenced by self-selection bias, with individuals who were already interested in or supportive of F-words more likely to respond; however, these participants provided important critiques, constructive ideas and balanced feedback. Additionally, we do not have information regarding the number of eligible participants across sites to estimate an adequate survey response rate, as this was a pilot study. Thus, generalizability of findings should be approached cautiously. Further, transferability of qualitative findings should be considered. Conclusions apply to our specific sample, and due to potential self-selection bias, SP roles, and participant gender imbalance (high percentage of women participants), transferability should be assessed based on the new context in which these findings are being considered. Finally, since the Likert-scale questions were self-developed, their psychometric properties are unknown, and responses reflect self-reported perceptions rather than validated constructs.

### 4.2 Future research

Since this was part of a pilot study, future research should build on these findings and continue to explore F-words implementation with new/diverse sites. Researchers may also engage SPs and organizations that are not yet using the F-words, to better understand barriers to uptake and reasons for non-adoption. Including SPs from a broader range of contexts (e.g., social services, education, diverse cultural communities, international settings) will be essential to examine how contextual and sociocultural factors influence F-words use. Ongoing work related to cultural adaptations of the F-words highlights the need for deeper exploration of how cultural values, beliefs and social and structural environments shape both perceptions of the framework and the ways SPs integrate it into practice. There is a need for more culturally and socially responsive implementation strategies. One project, funded through the Canadian Institutes of Health Research Health System Impact Program,^6^ has begun to explore and address these gaps.

## 5 Conclusion

This study provides novel insights into how SPs across five sites are using F-words, and their perceived impact of the F-words. Overall, the findings demonstrate that while the F-words are generally well-received and viewed as beneficial, intentional strategies may be needed to strengthen organizational implementation efforts and supports, build SP confidence, and promote consistent use in safe and meaningful ways. For organizations, investing in leadership support, staff training, adequate resources, and building implementation infrastructure may promote F-words use. Since this was a pilot study, it is important to continue exploring barriers and facilitators to implementing F-words and useful implementation strategies across diverse settings. This study provides an important foundation for researchers to continue exploring how the F-words may be integrated into pediatric rehabilitation and related sectors and the perceived impacts on SPs and children with NDDs and their families.

## Data Availability

The raw data supporting the conclusions of this article will be made available by the authors, without undue reservation.

## Footnotes

1 CanChild’s F-words Knowledge Hub: https://canchild.ca/research-in-practice/f-words-in-childhood-disability/.

2 Tomas et al. Implementing the ‘F-words for Child Development’: Learnings from Health and Developmental Services Organizations in Canada and New Zealand. *Global Implementation Research and Applications.* Under Review.

3 F-words Online Training Program: https://canchild-fwords.ca/.

4 F-words Trainings: https://canchild.ca/research-in-practice/f-words-in-childhood-disability/f-words-training/.

5 What If Tool: https://canchild.ca/wp-content/uploads/2026/01/What-If-Guide-Complete-Jan-2026-ACCESS.pdf.

6 Health Systems Impact Program 2023 Projects: https://cihr-irsc.gc.ca/e/53687.html.
