# Supplementary material for: Service provider perspectives on implementing the ‘F-words for child development’: an innovative approach to children’s health and developmental services
Source: Front Public Health. 2026 Sep 18;14:1788060. doi: 10.3389/fpubh.2026.1788060 (PMC13630929; doi:10.3389/fpubh.2026.1788060)
Supplement: Supplementary file 1 [file Supplementary_file_1.DOCX]

**Supplementary Material 1**

**Service Provider Interview Guide**

**Questions:**

1. First off, thank you again for agreeing to do this interview. Can you begin by telling me more about yourself?
   - *Probes: Name, the organization where you provide services, your current role, people you work with.*
2. What do the F-words mean to you?
   - *PROBES can be around what they mean in the context of their life, the services they provide, and the families they work with*
   - *QUESTION: Do you like them, dislike them, unsure?*
   - *QUESTION: Do you believe there is a need for the ‘F-words’? Why or why not?*
3. Who have you used the F-words with? (e.g., parents, children, youth, colleagues)
   - *QUESTION:* How did you use the F-words with these people?
   - *QUESTION:* Are there other people at your organization who you would like to see use the F-words?
   - *QUESTION:* How would you like to see the F-words continue to be used at your organization?
4. Has your relationship with families changed since using the F-words?
   - *PROBES can be around interactions with each other, trust, learning more about the family, prioritizing family goals / story (i.e., family-centredness)*
5. What do you think the impact of the F-words is on the services within your organization?
   - *PROBES can be around what they’ve noticed among the families they work with (e.g., engagement, how families talk about their child, specific services), how they approach service-delivery and how this is different from what they did before, and their thoughts and perceptions of child health and abilities*
6. What helped you use F-words in your practice?
   - *PROBES can be around supports they received, prompts, tools, etc.*
   - *QUESTION:* Did you experience any challenges with using the F-words?
7. Do you have any additional comments and/or feedback that you would like to add?
